# Impact of Immunotherapies on SARS-CoV-2-Infections and Other Respiratory Tract Infections during the COVID-19 Winter Season in IBD Patients

**DOI:** 10.1155/2022/3469789

**Published:** 2022-08-24

**Authors:** Constanze Heike Waggershauser, Cornelia Tillack-Schreiber, Paul Weyh, Eckard Alt, Thorsten Siegmund, Christine Berchthold-Benchieb, Daniel Szokodi, Fabian Schnitzler, Thomas Ochsenkühn

**Affiliations:** ^1^IBD Center Munich, Sonnenstraße 29, 80331 Munich, Germany; ^2^Synesis Research, Sonnenstraße 29, 80331 Munich, Germany; ^3^Isarklinikum, Sonnenstraße 24-26, 81244 Munich, Germany; ^4^Ludwig-Maximilians-University of Munich, Marchioninistraße 15, 81377 Munich, Germany

## Abstract

**Background:**

COVID-19 represents one of the most significant medical problems of our time.

**Aims:**

This study is focused on the question whether patients with inflammatory bowel disease (IBD) who receive immunotherapies are more vulnerable to respiratory tract infections and SARS-CoV-2 infections in comparison to medical staff, as a cohort with an increased infection risk, and to the general population in a COVID-19 hotspot.

**Methods:**

We analysed data regarding respiratory tract infections that were collected in our IBD registry and compared them with corresponding data from medical employees in our associated Isarklinikum hospital and from the healthy general population in Munich, Germany, over the same time frame in April and June 2020. Patients were tested for SARS-CoV-2 immunoglobulins (Ig).

**Results:**

Symptoms of respiratory tract infections occurred equally frequent in IBD patients with immunotherapies as compared to those without. Older age (>49 years), TNF-inhibitor, and ustekinumab treatment showed a significantly protective role in preventing respiratory tract symptomatic COVID-19 infections that occurred in 0.45% of all our 1.091 IBD patients. Of those, 1.8% were positive for SARS-CoV-2 Ig, identically to the general population of Munich with also 1.8% positivity. Whilst more than 3% of all COVID-19 subjects of the general population died during the first wave, none of our IBD patients died or needed referral to the ICU or oxygen treatment.

**Conclusions:**

In our study, IBD patients are as susceptible to respiratory tract infections or SARS-CoV-2 as the normal population. There is no evidence of an association between IBD therapies and increased risk of COVID-19. Interestingly, a reduced rate of COVID-19 deaths in IBD patients, the majority on immunomodulator therapy, was observed, compared to the general population. Therefore, no evidence was found to suggest that IBD medication should be withheld, and adherence should be encouraged to prevent flares. In addition to older age (>49 years), TNF inhibitors and ustekinumab show a protective role in preventing respiratory tract infections. In addition, these results add to the growing evidence that supports further investigation of TNF inhibitors as a possible treatment in the early course of severe COVID-19.

## 1. Introduction

Coronavirus disease 2019 (COVID-19), caused by the severe acute respiratory syndrome coronavirus-2 (SARS-CoV-2), developed into a pandemic and was declared a global emergency by the WHO in early 2020 [1, 2]. In Germany, the first confirmed case of COVID-19 was registered on January 27th of 2020 [3] and the virus spread to 13 of 16 federal states within one month [4]. In Germany, the exponential increase in newly confirmed cases during the first wave had reached a total of 155,193 cases on April 27, 2020 (187 per 100,000 inhabitants over a period of 7 days) with a hotspot in Munich. In May 2020, the symptomatic infection rate was 0.46%, and a mortality of 3.4%, particularly amongst old and sick patients, was recorded [5].

Susceptibility to SARS-CoV-2, a potential greater risk of developing COVID-19 and an increased risk of progression to severe disease courses or even death compared to the general population, was, and still is, a concern among physicians and patients with inflammatory bowel disease (IBD). Background of this concern is the increased risk of infections due to immunotherapies and the expression of the receptor angiotensin-converting-enzyme (ACE) 2 and the transmembrane serine protease (TMPRSS) 2, which mediates SARS-CoV-2 entry into the cells in the esophagus and enhanced in about 30% in the small intestine. In more than 11% of confirmed COVID-19 cases, patients presented gastrointestinal symptoms, such as nausea, vomiting, or diarrhoea. Physiopathologically, the GI tract can be affected by direct viral infection of the esophageal or intestinal epithelium, secondary by viremia following lung infection. Further, ACE2 is involved in the regulation of the intestinal microflora [6]. ACE2 and TMPRSS are significantly upregulated in IBD with mucosal inflammation [7, 8] and therefore may even increase host susceptibility. Meanwhile, international cohorts described no increased risk of symptomatic COVID-19 for patients with IBD [9, 10].

As numbers of asymptomatic or minimally symptomatic SARS-CoV-2 carriers are unknown, we only have estimates on actual numbers of SARS-CoV-2 infections [11–13]. In addition, the numbers of confirmed cases depend on access to healthcare, laboratory availability, and the criteria applied to select the individuals who should be tested [14]. Therefore, the real infection rates, as well as rates of hospitalization and mortality, remain to be established in IBD patients. The analysis of patient cohorts can lead to reliable results and allow choosing the best therapy in times of the SARS-CoV-2 pandemic.

According to the suggestions of the European Crohn's and Colitis Organization (ECCO) in March 2020 [15], we continued our regular ambulatory service for all IBD patients and started an observational study. We administered immunosuppressants, immunomodulating therapies peroral, intravenous, or subcutaneous (p.o., i.v., or s.c.) and even started new immunotherapies. Only appointments of patients who felt unwell, reported fever and cough, or had contact with SARS-CoV-2 positive patients were postponed for two weeks.

Recently, we described a part of our survey of our IBD cohort [16], showing that the frequency of respiratory tract infections was not increased, nor was the frequency of COVID-19. Now, we extended our cohort characterization by looking at the type of immunomodulation. We determined the prevalence of SARS-CoV-2 antibodies in our IBD cohort and compared the results with PCR-proven symptomatic SARS-CoV-2 infections. Data were related to those from healthcare workers in our associated hospital as an independent COVID-19 risk group. In addition, our IBD data were compared to those of the general population in the city of Munich during the same time period in the first half year of 2020 describing 0.49% symptomatic COVID-19-positive residents in 2,994 representative private households and seropositivity for SARS-CoV-2 in 1.8% [14].

### 1.1. Materials, Methods, and Statistics

From April 1st until May 31st, 2020, 1,200 patients of our IBD center were contacted. As reported by the ethics committee [17], 1,091 successfully completed the questionnaire and 777 of them agreed in serum analysis. Since the actual estimated number of silent SARS-CoV-2 infections was probably high and most infected individuals present with only light or moderate symptoms of respiratory tract infection, we asked patients for any of the following symptoms since the start of the pandemic: cough, rhinitis, sore throat, fever and chills, dyspnea, loss of smell (anosmia), or loss of taste (ageusia). In addition, we asked for the results of SARS-CoV-2 RNA swab tests and IBD-related information.

In those 777 patients, who agreed to antibody testing, we took blood for measuring immunoglobulins against SARS-CoV-2 offered between April 9 and June 30, 2020. If a symptomatic respiratory tract infection was recalled, blood was taken at least three weeks after this infection.

Blood plasma was extracted from whole blood and frozen at −20°C until immunoglobulin (Ig) measurement. To determine the presence of SARS-CoV-2 Ig, we used the Elecsys^®^ Anti-SARS-CoV-2 immunoassay (Roche Diagnostics Deutschland GmbH, Mannheim) against the nucleocapsid antigen. The test showed a sensitivity of 99.5% (95% CI 97–100%) more than 14 days after an infection and specificity of 99.81% (95% CI 99.67–99.90%) in a routine panel. According to the manufacturer's manual, Ig are still measurable more than 60 days after infection with a very high sensitivity.

In a second analysis, we asked health care workers (physicians, nurses, and other personnel) of our associated Isarklinikum hospital in Munich to complete the same questionnaire [16] and to agree to serum analysis. Written consent from all patients and individuals who completed the questionnaire and gave blood for the study analysis was obtained.

Respiratory tract infection rates in the general population were taken from a prospective study of the University of Munich that covered the same period. It has recently been published by Pritsch et al. [14] and provided a detailed description of the SARS-CoV-2 infections and COVID-19 in Munich, Germany, between April and June 2020: 2,994 representative private households with 5,313 participants completed questionnaires and provided blood samples. Symptomatic COVID-19 diseases were found in 0.49% of the general population at the end of this first disease wave. Seropositivity for SARS-CoV-2 specific antibodies was 1.82%.

For statistical analyses, we first used the Shapiro–Wilk test to test a Gaussian distribution of our data. If *p* > 0.1, we went on with the *t*-test, if not, the Mann–Whitney *U* test was used. To compare the frequency distribution, the Chi-squared test or Fisher's exact test was applied. For investigation of factors that could have an influence on the occurrence of symptoms of upper respiratory tract infections, logistic regression models were used.

## 2. Results

### 2.1. Characterization of the IBD Study Cohort

Of 1,091 IBD patients completing the questionnaire between April and June 2020, 53.4% (*n* = 583) had Crohn's disease (CD), 46.6% (*n* = 508) had ulcerative or indeterminate colitis (UC), 54.8% (*n* = 598) were female, and 45.2% (*n* = 493) were male. The median age was 41.9 years, ranging from 18 to 89 years, and 68.8% of all patients were in remission as defined by the respective scores CDAI for CD and MAYO for UC. 70.3% (*n* = 767) of all patients were 18 to 49 years old, and 29.7% (*n* = 324) of patients were older. The median disease duration among all patients was 146.8 months, ranging from 0.5 to 650 months. Immunotherapies were given in 77.2% of patients (*n* = 842) and 22.8% of patients (*n* = 249) were not receiving immunotherapies (Table 1).

Primary statistical analysis showed no differences in age or disease activity between the respective patient groups with and without immunotherapies, but patients with immunotherapies had more often CD (*p* > 0.001), and patients with no immunotherapies had more often UC (*p* < 0.001). Patients with immunotherapies were more often male (*p*=0.003) and had longer disease duration than patients without immunotherapy (*p*=0.048). More patients with no immunotherapies were female, as compared to female patients with immunotherapies (*p*=0.003, Table 2). Distribution of age, sex, disease, disease duration, and activity within the respective immunomodulatory medication groups can also be found in Table 2.

Distribution of age sex, disease, disease duration, and activity among respective and immunomodulatory status within the older (>49) and the younger (18–49) patient groups are shown in Table 3.

### 2.2. Symptoms of Respiratory Tract Infections among IBD Patients with and without Immunotherapies

Any symptoms of respiratory tract infections occurred less frequently in patients with immunotherapy (34.8%) than in patients without (42.6%, *p*=0.025).

**Figure 1:**
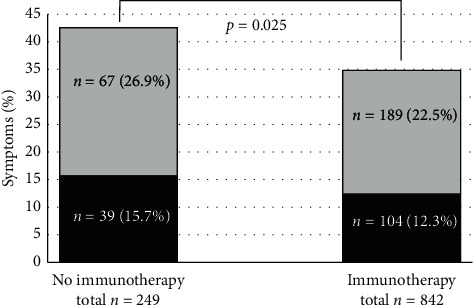
Symptoms of respiratory tract infections: moderate in grey (cough, rhinitis, and sore throat) and severe in black (fever, chills, and anosmia) in patients with or without immunotherapies. Patients with immunotherapies show significant less respiratory symptoms (*p*=0.025).

When differentiating between moderate symptoms of respiratory tract infection (like coughing, rhinitis, or sore throat) and severe symptoms (like fever, chills, or anosmia), neither moderate nor severe symptoms occurred more frequently in patients with immunotherapy than in patients without (moderate: 12.4% vs 15.7%, *p*=0.174 and severe: 22.5% vs 26.9%, *p*=0.079, Figure 1).

To investigate the factors influencing the occurrence of those symptoms, we applied logistic regression analysis for the following predictor variables: immunotherapy, type of IBD (UC or CD), age, sex, age at diagnosis, and remission status, respectively.

After including all variables, regression analysis identified immunotherapy and older age as the only significant factors protecting from the occurrence of symptoms of upper respiratory tract infections:

- Immunotherapy: Odds ratio (OR) 0.713 (95% CI [0.52; 0.978], *p*=0.036).
- Older age: OR 0.977 (95% CI [0.965; 0.988], *p* < 0.001).

Symptoms of respiratory tract infections among IBD patients with specific immunotherapies were compared to patients without immunotherapies.

**Figure 2:**
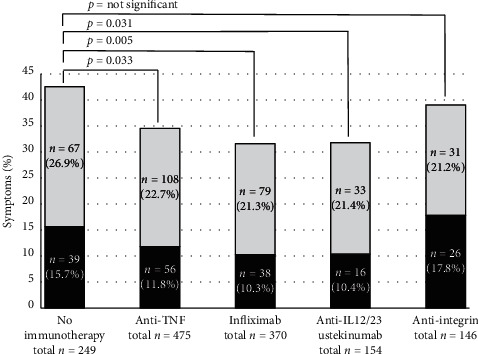
Symptoms of respiratory tract infections in IBD patients: moderate in grey (cough, rhinitis, and sore throat) and severe in black (fever, chills, and anosmia), in patients without immunotherapy or according to their immunotherapies. Patients with anti-TNF, infliximab, and ustekinumab treatment show significant less respiratory symptoms compared to those with no immunotherapies (*p* < 0.05).

Subgroup analyses were performed for the major immunotherapies; overall symptoms of respiratory tract infections were significantly less frequent in patients treated with ustekinumab (31.8%, *p*=0.031), infliximab (31.6%, *p*=0.005), and all TNF inhibitors (infliximab, adalimumab, and golimumab; 34.5%, *p*=0.033) as compared to patients without immunotherapies (42.6%). No difference was seen in the vedolizumab group (*p*=0.492) (Figure 2).

To investigate the factors influencing the occurrence of symptoms among patients within the different immunotherapy groups, we again applied logistic regression analysis for the following predictor variables: type of immunotherapy (all TNF inhibitors, infliximab, or ustekinumab), age, age at diagnosis, and remission status, respectively .

Receiving either TNF-blockers, infliximab, or ustekinumab, respectively, and age were the only significant factors protecting from the occurrence of symptoms of upper respiratory tract infections:

- All TNF-blockers: OR 0.656 (95% CI [0.46; 0.936], *p*=0.020).
- Infliximab: OR 0.572 (95% CI [0.39; 0.831], *p*=0.003).
- Ustekinumab: OR of 0.631 (95% CI [0.38; 0.991],*p* < 0.046).

Symptoms of respiratory tract infections among young and old IBD patients with and without immunotherapies are compared.

**Figure 3:**
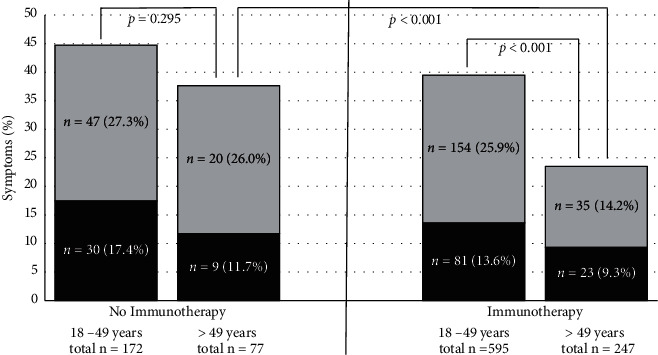
Symptoms of respiratory tract infections: moderate in grey (cough, rhinitis, and sore throat) and severe in black (fever, chills, and anosmia) in patients without (left) or with immunotherapy (right) according to the patients' age. Old patients with immunotherapy show less symptoms than old patients without immunotherapy (*p* < 0.001). Further, old patients with immunotherapy show less symptoms than young patients with immunotherapy (*p* < 0.001). Older IBD patients without immunotherapy had not more symptoms than younger IBD patients without immunotherapies (*p*=0.295).

To clarify the influence of age on symptoms of respiratory tract infections, we compared IBD patients older than 49 years to patients younger than 50 years with and without immunotherapies:

- Older IBD patients had less frequent symptoms than younger IBD patients (26.9% vs 40.7%, *p* < 0.001).
- Older IBD patients without immunotherapies had not more symptoms than younger IBD patients without immunotherapies (37.7% vs 44.8%, *p*=0.295)
- Older IBD patients with immunotherapies had less symptoms than younger patients with immunotherapies (23.5% vs 39.5%, *p* < 0.001).
- Older IBD patients with immunotherapies had less symptoms than older IBD patients without immunotherapies (23.5% vs 37.7%, *p*=0.014) (Figure 3).

We therefore again applied two logistic regression analyses and found that in patients with immunotherapies age older than 49 years was protective against any respiratory tract infection symptoms (OR of 0.470, 95% CI [0.336; 0.659], *p* < 0.001). In the second analysis, we found out that in patients older than 49 years, immunotherapy is protective against any respiratory tract infection symptoms (OR of 0.508; 95% CI [0.294; 0.877], *p*=0.015).

### 2.3. Characterization of the Health Care Workers Including Symptoms of Respiratory Tract Infections

In the tertiary Isarklinikum hospital, 435 health care professionals (nurses, doctors) completed the questionnaire. The median age was 38 years (17–70), 339 (78%) were female, and 348 (80.0%) of those employees were 18 to 49 years old, whilst 87 (20.0%) were 50 to 89 years old.

Among all 435 study participants, overall symptoms of respiratory tract infections occurred in 155 medical employees. Of all 155 participants with symptoms, 78% (*n* = 121) were female, and 85% (*n* = 132) were 18 to 49 years old. 90 (20.7%) presented with only mild and moderate symptoms like coughing, rhinitis, or sore throat. Severe symptoms of respiratory tract infections like fever, chills, and anosmia occurred in 65 participants (14.9%).

### 2.4. Prevalence of SARS-CoV-2 infections and the Development of COVID-19 in the IBD Cohort

Of all 1,091 patients of our IBD study, who completed the questionnaire, 58 had received a SARS-CoV-2 rt-qPCR swab test due to respiratory symptoms. Of those, five (0.45%) patients were SARS-CoV-2 positive; all of them presented with severe symptoms.

In addition, in 777 patients in our study from whom also blood was taken, we found the serological signs of an infection with SARS-CoV-2 by detecting anti-SARS-CoV-2 immunoglobulins in 14 patients (1.8%). Those 14 included the five positive swabs and identified another nine patients, in whom no swab test had been performed, because no COVID-19 had been suspected by the patients or their physicians. Of those 14 patients, 7 had CD and 7 were female; their median age was 40.5 years (29–79), 11 received biological therapies, of which eight consisted of TNF inhibitors, one of ustekinumab, and two of vedolizumab. The remaining three patients were treated with oral mesalazine and/or budesonide (Table 4). None of our 14 infected patients needed oxygen therapy or had to be admitted to a hospital; eight patients suffered from only moderate symptoms of respiratory tract infection; and one patient recalled only fatigue. Five suffered from severe symptoms of respiratory tract infection. They all were home quarantined, and their disease-course remained uneventful, and immunotherapies were continued.

### 2.5. Prevalence of SARS-CoV-2 Infections and the Development of COVID-19 in Hospital Employees

All participating 435 hospital employees provided blood for SARS-CoV-2 Ig analysis. Of those, 13 (2.99%) employees were positive; all of them in the age between 18 up to 49 years and 7 (53.8%) were female. Six of those 13 reported no symptoms, one reported moderate, and six reported severe symptoms. In five of those 13, no swab test had been performed because no COVID-19 had been suspected by the patients or their physicians.

Of all participants, 17 (3.9%) were taking immunosuppressives and only one of them got infected with SARS-CoV-2. No participants needed oxygen therapy or had to be admitted to a hospital; all disease courses remained uneventful.

## 3. Discussion

In this study, we made three major observations. First, we found that IBD patients showed a comparable rate of SARS-CoV-2 seropositivity (1.8%) as the local general population without a higher mortality or morbidity from COVID-19. Second, we could show that non-COVID respiratory tract infections occurred equally frequent in IBD patients with immunotherapies as compared to those without. And third, we saw that among those IBD patients, older age (>49 years) or taking TNF inhibitors, especially infliximab or ustekinumab, protected from respiratory tract infections.

Further, we show that a questionnaire can collect exact medical data and discriminate between mild and severe symptoms without a face-to-face contact between doctor and the patient. Potentially, this can be a start of a remote patient monitoring in our IBD cohort. For example, Sinagra and colleagues already showed that telemedicine in IBD patients is very well accepted and an effective tool in monitoring the disease activity in quiescent and mild IBD [18], even subsequent COVID-19. Remote patient monitoring (e.g., phone calls, video consultations, and questionnaires) not only reduce potential infection transmission risks but also enable more patient contacts in an increasing IBD patient cohort worldwide.

To our actual knowledge about the SARS-CoV-2, early events in the pathogenesis of SARS-CoV-2 infection include attachment of the viral spike protein to epithelial ACE2 and cleavage by TMPRSS2, which facilitates viral entry into the cytoplasm of the host cell [19]. Since intestinal inflammation upregulates the ACE2 receptor and the TMPRSS2 protease, IBD patients may be at an increased risk for SARS-CoV-2-infection and might be prone to a more severe disease course. Further, the presence of autoantibodies (antinuclear antibodies (ANA), anticardiolipin antibodies, and anti-*β*2-glycoprotein antibodies) is elevated in COVID 19 hospitalized patients. The most prominent are nuclear ANAs in 25 up to 50%, often seen in IBD patients too. Patients tested positive for autoantibodies had a significantly more severe prognosis than others [20, 21]. It may be that autoantibodies are reflecting a pathogenetic role of immune dysregulation. Since the patients' numbers are small (12 to 64 patients per study) and the methods of antibody detection are different, no clinical consequence can be concluded so far.

Besides this, the therapeutic armamentarium for patients with IBD includes immunosuppressives, like purine analogues or methotrexate, and immunomodulators, like TNF inhibitors, and non-TNF-targeted biologics and targeted small-molecule therapies [22]. However, these therapies could weaken the immune system and potentially place IBD patients at increased risk of infections and infectious complications. Consequentially, there is a concern that IBD patients are at greater risk of infection with SARS-CoV-2 and at increased risk of progressing to a more severe clinical course or even death compared to the general population. In addition, if an IBD patient develops COVID-19, there was a lack of guidance on medication management.

Our data did not reveal any evidence that IBD patients who receive immunotherapies have an increased risk for respiratory tract infections or a higher severity if they get infected. Of note, older patients seem to show reduced signs overall. Therefore, unlike commonly suspected for many years [23, 24], we found no evidence that immunotherapies expose older people to a higher risk of symptomatic respiratory tract infections. This observed phenomenon of a potential beneficent influence of immunotherapies among older patients could lead to further studies in geriatric infectiology.

Further, our studied IBD patients showed an incidence rate of symptomatic SARS-CoV-2 infections (0.45 and 0.49%) that was comparable with the general population [5] in the same city within the same time frame in 2020. The course of SARS-CoV-2 infections in all IBD patients (receiving immunotherapies or not) was mild and uneventful. None of our IBD patients needed oxygen therapy, respiratory therapy, or ICU treatment. There was no difference in the prevalence and disease course between patients with or without immunotherapy. None of our patients died or needed hospitalization, oxygen, or ICU treatment. A small cohort of IBD patients who is under medical surveillance cannot fully be compared with the general population regarding mortality. However, the fact that 3.4% of patients with COVID-19 died in the general population in Munich [5] during the same time does at least shed a positive light on the impact of immunotherapies. Therefore, our data provide additional support that immunotherapies should not be stopped or delayed during the COVID-19 crisis. Immunotherapy did not increase infection rates, neither in young and old patients, nor did it attenuate the disease course of COVID-19 to the worse. This is in accordance with the observational results of the Surveillance Epidemiology of Coronavirus under Research Exclusion for IBD (SECURE-IBD) registry [25]. 7,038 COVID-19 cases were reported until March 26, 2022, with diverse disease activities, comorbidities, and therapies. In the SECURE-IBD registry, lowest numbers of hospitalization, ICU treatment, or death of IBD patients are described in patients with TNF-inhibitor treatment.

Further, two other large worldwide registries of individuals with immune-mediated inflammatory diseases have demonstrated an inverse association of anti-TNF-alpha use and the severity of COVID-19-related outcomes [26, 27]. TNF-alpha is a proinflammatory cytokine that is intimately involved in excess inflammation. An early increase and high levels of TNF-alpha in COVID-19 predicts mortality [28–30]. Furthermore, TNF-alpha is involved in key features of COVID-19 with poor outcome: elevated TNF-alpha levels have been associated with increased capillary leaking and the risk of thrombosis in ARDS [31, 32] and the action of neutrophils [33]. Karki et al. describe the use of neutralizing TNF-alpha- and IFG-antibodies in a mouse model of SARS-CoV-2; the combination increases the survival from 14 up to 50% [34]. Stallmach et al. retrospectively collected data on the rescue use of anti-TNF in patients suffering severe COVID-19 and they found a reduced mortality [35] with only one of seven infliximab- (IFX-) treated patients dying as compared to one-third of 17 patients without IFX.

Suárez-Fariñas et al. [8] describe multiple gene sets with a significant overlap between COVID-19 response genes and genes associated with active IBD. Further, they observed that key driver genes, which were upregulated with COVID-19 and IBD inflammation (e.g., CXCL1, GBP4, SOCS3, PARP14, and PARP9), were downregulated following the use of infliximab. In addition, anti-TNF-alpha was able to reduce the enteric expression of ACE2 and TMPRSS [8].

In conclusion, IBD patients do not appear to be more susceptible or vulnerable to SARS-CoV-2 infections or other respiratory tract infections. There is no evidence of an association between IBD therapies and an increased risk of COVID-19 and IBD therapies should not be stopped nor delayed. Being older than 49 years and taking TNF blockers or ustekinumab might indicate a protective role in preventing respiratory tract infections.

Our data support further investigation of the use of anti-TNF agents in the early time course of patients with severe COVID-19.

## Figures and Tables

**Table 1:** Characteristics of the IBD cohort.

	Ulcerative colitis or Crohn's disease *n* (%)
Total number	1091 (100)
Patients 18 to 49 years	767 (70.3)
Patients older than 49 years	324 (29.7)
Female	599 (54.9)
No immunotherapies	249 (22.8)
Steroids only	8 (0.7)
TNF-alpha blockers: infliximab, adalimumab, golimumab	475 (43.5)
Infliximab	370 (33.9)
Adalimumab	95 (8.7)
Golimumab	9 (0.8)
IL 13/23 antibody: ustekinumab	154 (14.1)
Anti-integrin: vedolizumab	146 (13.4)
Immunotherapy combinations	45 (4.1)
Others (tofacitinib, purine analogues, methotrexate)	14 (1.3)

**Table 2:** IBD cohort characteristics by therapy.

	Patients without immunotherapy *n* (%)	Patients with immunotherapy *n* (%)	TNF-alpha blockers: infliximab, adalimumab, golimumab *n* (%)	Infliximab *n* (%)	Adalimumab *n* (%)	Ustekinumab *n* (%)	Vedolizumab *n* (%)	Immunotherapy combinations *n* (%)
Total number	249 (22.8)	842 (77.2)	475	370	95	154	146	45
CD	104 (41.8)	479 (56.9)	313 (65.9)	233 (63.0)	76 (83.2)	102 (66.2)	34 (23.3)	26 (57.8)
UC	145 (58.2)	363 (43.1)	162 (34.1)	137 (37.0)	16 (16.8)	52 (33.8)	112 (76.7)	19 (42.2)
Female	157 (63.1)	442 (52.5)	225 (47.4)	116 (45.1)	53 (55.8)	97 (63.9)	84 (57.5)	22 (48.9)
Median age	43.1 years, range 19–88	41.6 years, range 18–89	41.2 years, range 18–87	40.8 years, range 19–87	41.9 years, range 18–77	43.5 years, range 21–83	43.1 years, range 19–89	33.8 years, range 20–69
Median disease duration	140.5 months, range 0.5–580	148.6 months, range 0.5–650	141.6 months, range 0.5–650	135.1 months, range 0–650	169.5 months, range 10–471	168.0 months, range 10–556	150.5 months, range 10–448	160.8 months, range 7–519
Age >49 years	77 (30.9)	247 (29.3)	128 (27.0)	98 (26.5)	25 (26.3)	50 (32.5)	39 (26.7)	3 (6.7)

**Table 3:** Patients' characteristics of young versus old patients.

	Patients 18–49 years *n* (%)	Patients >49 years *n* (%)
Total number	767 (70.3)	324 (29.7)
CD	412 (53.7)	171 (52.8)
UC	355 (46.3)	153 (47.2)
Female	420 (54.8)	178 (54.9)
No immunotherapy	172 (22.4)	77 (23.8)
With immunotherapy	595 (77.6)	247 (76.2)
TNF-alpha blockers: infliximab, adalimumab, golimumab	347 (45.2)	128 (27.0)
Infliximab	272 (35.5)	98 (26.5)
Adalimumab	70 (9.1)	25 (26.3)
Ustekinumab	104 (13.6)	50 (32.5)
Vedolizumab	107 (14.9)	39 (26.7)
Immunotherapy combinations	42 (5.5)	3 (6.7)

**Table 4:** Patients' characteristics with positive SARS-CoV-2 Ig tests. Patients in bold were swab test positive.

Patients	Age in years	Sex	Disease	Therapy	Symptoms	Travel history
A. K.	30	Female	CD	Infliximab every 8 weeks i.v.	Cough, rhinitis	Sri Lanka 03/2020
A. S.	53	Male	CD	Infliximab every 8 weeks i.v.	Headache, limb pain, anosmia	
E. M.	49	Male	CD	Budesonide p.o.	Sore throat, fever	Austria 03/2020
M. C.	32	Female	CD	Infliximab every 8 weeks i.v.	Cough, rhinitis, headache, dyspnea	Egypt 03/2020
P. C.	45	Female	CD	Infliximab every 6 weeks i.v.	Cough, limb pain, dyspnea, abdominal pain and shingles	
R. C.	29	Female	CD	Adalimumab 40 mg every 2 weeks s.c.	Cough, rhinitis, sore throat, abdominal pain	Austria 03/2020
W. F.	46	Male	CD	Ustekinumab 90 mg every 8 weeks s.c. plus 50 mg azathioprine daily p.o.	Cough, rhinitis, sore throat, abdominal pain	Netherlands 02/2020
B. P.	45	Male	UC	Mesalazine daily p.o.	Cough, rhinitis, sore throat	Austria 02/2020
C. M.	29	Female	UC	Adalimumab 80 mg every 2 weeks s.c.	Cough, chills	
R. V.	29	Female	UC	Vedolizumab 300 mg every 8 weeks i.v.	Cough, sore throat, chills	
R. R.	36	Male	UC	Infliximab every 8 weeks i.v.	Cough, sore throat	
S. M.	36	Male	UC	Vedolizumab 300 mg every 8 weeks i.v.	Cough, rhinitis, expectoration	
S. V.	79	Male	UC	Infliximab every 8 weeks i.v.	Fatigue, loss of wight	
W. M.	29	Female	UC	Mesalazine daily p.o.	Cough, rhinitis, fatigue	

## Data Availability

The data that support the findings of this study are available upon request from the corresponding author.
